# Three-dimensional catheter tip force sensing using multi-core fiber Bragg gratings

**DOI:** 10.3389/frobt.2023.1154494

**Published:** 2023-03-09

**Authors:** Omar Al-Ahmad, Mouloud Ourak, Johan Vlekken, Eric Lindner, Emmanuel Vander Poorten

**Affiliations:** ^1^ Robot-Assisted Surgery (RAS) group, Department of Mechanical Engineering, KU Leuven University, Leuven, Belgium; ^2^ FBGS International NV, Geel, Belgium

**Keywords:** catheter ablation, fiber Bragg gratings, force sensor, miniaturized sensor, multi-core fiber

## Abstract

Awareness of catheter tip interaction forces is a crucial aspect during cardiac ablation procedures. The most important contact forces are the ones that originate between the catheter tip and the beating cardiac tissue. Clinical studies have shown that effective ablation occurs when contact forces are in the proximity of 0.2 N. Lower contact forces lead to ineffective ablation, while higher contact forces may result in complications such as cardiac perforation. Accurate and high resolution force sensing is therefore indispensable in such critical situations. Accordingly, this work presents the development of a unique and novel catheter tip force sensor utilizing a multi-core fiber with inscribed fiber Bragg gratings. A customizable helical compression spring is designed to serve as the flexural component relaying external forces to the multi-core fiber. The limited number of components, simple construction, and compact nature of the sensor makes it an appealing solution towards clinical translation. An elaborated approach is proposed for the design and dimensioning of the necessary sensor components. The approach also presents a unique method to decouple longitudinal and lateral force measurements. A force sensor prototype and a dedicated calibration setup are developed to experimentally validate the theoretical performance. Results show that the proposed force sensor exhibits 7.4 mN longitudinal resolution, 0.8 mN lateral resolution, 0.72 mN mean longitudinal error, 0.96 mN mean lateral error, a high repeatability, and excellent decoupling between longitudinal and lateral forces.

## 1 Introduction

### 1.1 Medical overview

Cardiovascular diseases (CVDs) are the leading cause of global mortality (Roth et al., 2020). Coronary artery diseases, heart attacks and arrhythmias are the most common CVDs. Atrial Fibrillation (AFib) is a type of CVD that affects around 59.7 million patients globally (Li et al., 2022). A common treatment of AFib is through ablation and scarring of cardiac tissue with the aim of impeding occurrences of irregular contraction waves (Calkins et al., 2009). Atrial ablation is usually performed by minimally invasive catheterization approaches (Lairikyengbam et al., 2003). In a consensus paper, Calkins *et al.* established the superior safety and efficacy of catheter-based approaches compared to open surgical approaches (Calkins et al., 2012). Although minimally invasive, catheter ablation is not free of risks. When too large forces are applied, complications such as cardiac perforation, cerebrovascular accidents and atrio-esophaegeal fistula may arise (Sra, 2008; Tang, 2017). On the other hand, applying too low forces during ablation may lead to unsuccessful scaring of the cardiac tissue, which in turn, may require for a repetition of the procedure. Zhou *et al.* recommends a contact force between 0.1–0.4 N for effective ablation (Zhou et al., 2017). However, given that the friction force between the catheter and the access port is an order of magnitude larger than the force between the ablation tip and the cardiac tissue, it is physically impossible for the electrophysiologist to perceive this latter contact force (Song et al., 2018). In this regard, the intricacy of catheter ablation procedures calls for high resolution and accurate contact force sensors.

### 1.2 Prior works

Several contact force sensors have been developed in the past for cardiac ablation catheters. Polygerinos *et al.* worked on different force sensor designs based on light intensity modulation (LIM). The sensors were primarily comprised of one or more optical fibers that were assembled in the direction of a reflector that is attached to a flexure. In their latest work (Polygerinos et al., 2013), Polygerinos *et al.* developed a 4 × 24.5 mm (outer diameter × length) force sensor with three separate fibers that was able to measure three-dimensional forces. The reported root mean square (RMS) force estimation error for the longitudinal and lateral cases was 30 mN and 21 mN, respectively. The force resolution was reported to be lessbin10 mN. Following a similar approach, Noh *et al.* designed a 3.5 × 13 mm three-dimensional force sensor based on LIM, three separate fibers, and a CCD camera (Noh et al., 2016). The reported maximum force estimation error for the longitudinal and lateral cases was 65 mN and 205 mN, respectively. The main limitations of approaches based on LIM is that they are prone to light intensity fluctuations and phase discontinuities (Abushagur et al., 2014; He et al., 2014). This may significantly affect the measurements and subsequently the force estimations. Furthermore, in the work of Noh *et al.*, the force sensor was also observed to have notable longitudinal and lateral force coupling effects. The advantage of LIM based sensors, however, is that the light intensity is insensitive to the environmental temperature (Noh et al., 2016), meaning that temperature compensation is not necessary. An alternative approach to LIM relies on fiber Bragg grating (FBG) sensors inscribed within an optical fiber. Gao et al. developed a 2.6 × 11 mm three-dimensional force sensor based on four separate fibers with inscribed FBGs and parallel flexures (Gao et al., 2018). The sensor was reported to have RMS force estimation errors for the longitudinal and lateral cases of 7.94 mN and 13.6 mN, respectively. The sensor was also reported to have force resolutions for the longitudinal and lateral cases of 1.27 mN and 1.96 mN, respectively. The limitation of the sensor is that it involves a complex manufacturing and assembly procedure in addition to having potential FBG chirp failure which is caused by the non-uniform strain distribution on the adhesion zone (Li et al., 2020). Shi et al. developed a 4 × 18 mm two-dimensional force sensor based on four optical fibers with inscribed FBGs and a symmetric flexure, yielding a force resolution of 4.6 mN (Shi et al., 2018). The reported maximum force estimation errors, under constant temperature, was 250 mN. Besides being able to only measure lateral forces, another limitation of the force sensor was that it had bare fibers making it prone to breakage and unwanted interactions with the environment. Moreover, temperature compensation was inadequate yielding large force estimation errors with varying temperatures. Shin *et al.* designed a 2.3 mm outer diameter three-dimensional force sensor based on three separate FBG-inscribed fibers (Shin et al., 2018). The limitations of the force sensor are agian related to complex manufacturing and the potential for FBG chirping failure. Finally, Li *et al.* developed a 4 × 20 mm three-dimensional force sensor with five separate FBG-inscribed fibers and a miniature flexure (Li et al., 2020). The reported maximum force estimation error for the longitudinal and lateral cases was 24 mN and 15.6 mN, respectively. The reported force resolution for the longitudinal and lateral cases was 0.63 mN and 0.64 mN, respectively. Their force sensor displays a high performance and adequate temperature compensation. However, the limitations of the force sensor is that it uses too many optical fibers (i.e., five fibers), has a complex manufacturing and assembly process, and has relatively large dimensions. As can be seen, prior works utilize a combination of independent fibers in the design of the force sensor. This is usually done to decouple between lateral and longitudinal forces or to achieve temperature compensation. The reader is referred to (Polygerinos et al., 2010; Su et al., 2017) for an in-depth review on similar fiber optics based force sensors. Commercially, the most widely used catheters with tip contact force sensing are the Fabry-Perot interferometry-based TactiCath™ Quartz ablation catheter from Abbott (Chicago, IL, USA), and the magnetic transmitter/locator based Thermocool Smarttouch^®^ catheter from Biosense Webster (Irvine, CA, USA). Bourier *et al.* assessed the performance of both force sensors and found that they exhibit a mean force error of 11.8 mN and 58.8 mN, and a maximum force error of 49.0 mN and 294.2 mN, respectively (Bourier et al., 2016, 2017).

Note that the scope of force sensing, for medical applications, extends beyond catheter-based interventions. For example, various electrical-based sensors have been developed within minimally invasive surgery (MIS) to achieve tactile sensing of forceps and graspers (Bandari et al., 2020; Othman et al., 2022). Similarly, several force sensing concepts have been developed for needle tip force sensing (Liang et al., 2018). Note, however, that optical-based sensing techniques, and especially FBG-based techniques, are advantageous in comparison with other sensing methods as they comprise: 1) electromagnetic immunity, 2) miniature size, 3) high interrogation speed, 4) multiplexing capabilities, and 5) high mechanical strength (Taffoni et al., 2013; Shi et al., 2017).

### 1.3 Paper contributions and structure

**FIGURE 1:**
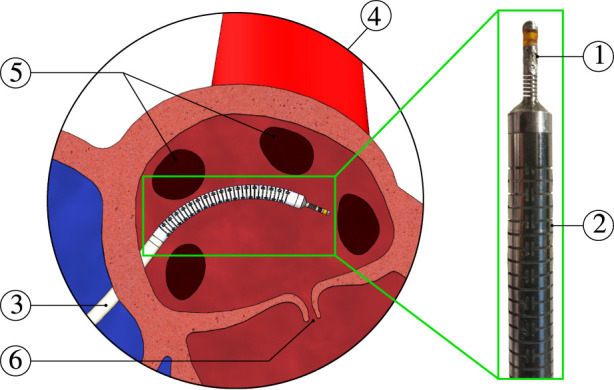
Catheter ablation scene: ① catheter tip force sensor, ② actuatable catheter, ③ medical delivery sheath, ④ zoomed-in view of the left atrium, ⑤ pulmonary veins, and ⑥ mitral valve.

In general, catheter tip force sensors may comprise functional or constructional limitations which include: non-linear measurement *versus* force output, force hysteresis, dimensional bulkiness, longitudinal and lateral force coupling effects, insufficient force estimation accuracy, and often complex designs or difficulty of assembly. In this paper, we propose the design of a novel catheter tip force sensor aiming to overcome all of the aforementioned limitations. The proposed sensor design is made to be simple, robust, and easily manufactured and assembled. The sensor design comprises a *single* multi-core fiber (MCF) with inscribed FBGs. This sensor embodiment avoids FBG chirping failure risks, fiber misalignments, and LIM-based limitations. The sensor embodiment also allows for redundant temperature compensation. To the best of the authors’ knowledge, this is the first implementation of a FBG-MCF for three dimensional force sensing. The developed force sensor is also integrated within an in-house built notched nitinol catheter that is actuatable through pneumatic artificial muscles (PAMs). This is done to prove the feasibility of integrating the force sensor within an actuatable catheter. It is important to note that the use of MCF technology not only allows for three-dimensional catheter tip force sensing, but also allows for a wide variety of other applications such as shape sensing (Moore and Rogge, 2012; Al-Ahmad et al., 2020), body force estimation (Aloi and Rucker, 2019; Al-Ahmad et al., 2021), and temperature monitoring. All of these capabilities can be integrated within a single catheter solution. This fits well with the targeted cardiac ablation application in this work (see Figure 1). The contributions of this paper are listed as follows.

• design and development of a novel catheter tip force sensor based on a multi-core fiber with inscribed FBGs,
• characterization and experimental validation of the force sensor using a dedicated experimental setup.

The rest of this paper is organized as follows: Section 2 covers theoretical design aspects including helical spring theory, FBG strain sensing, and combined MCF and spring modelling. Section 3 focuses on the methodology for decoupling between longitudinal and lateral forces. Section 4 discusses different aspects related to the calibration of the force sensor. Section 5 presents the calibration results, sensor performance, and corresponding discussions. Finally, concluding remarks and future work aspects are provided in Section 6.

## 2 Force sensor design

**FIGURE 2:**
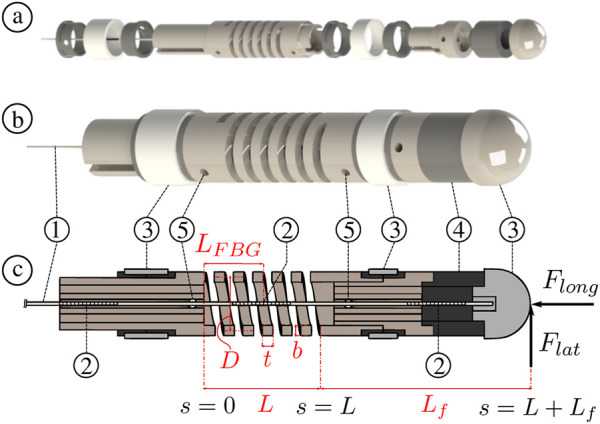
Force sensor overview ⓐ exploded view ⓑ assembled view ⓒ sectional view illustrating the relevant spring dimensions such as total length *L*, wire thickness *t*, wire breath *b*, and mean coil diameter *D*. The central FBG location along the spring’s length is given as *L*_*FBG*_. The sensor’s components include: ① MCF, ② FBGs, ③ ablation electrodes, ④ insulation ring, and ⑤ side holes to inject adhesives for MCF fixation.

The force sensor’s working principle is based on a single MCF inscribed with FBGs. A MCF differs from a single core fiber in that it contains a central core, but also circumferentially distributed outer cores at a given radial distance from the center (see Figure 4). All of the cores have FBGs inscribed into them meaning that strain can be sensed at different discrete locations along the MCF’s cross-section. The MCF is embedded within a helical compression spring which acts as the force-relaying flexure. The MCF is also rigidly attached to both ends of the spring to create a two-point fixation (see Figure 2). Temperature changes and external forces relayed to the MCF cause it to be strained internally. These internal strains are measured using the FBGs.

This section provides a theoretical approach towards the design and proper construction of the force sensor assembly. The theoretical approach provides the ability to customize and dimension the force sensor to achieve a user-defined performance. In this work, the force sensor is designed to achieve longitudinal and lateral force resolutions of ≤1 g (i.e., $\leq 9.81\times 10^{-3}$ N), which is the targeted performance metric.

### 2.1 Helical spring theory

#### 2.1.1 Longitudinal deflection

Figure 2C illustrates a sectional view of the force sensor with a helical compression spring having a total length *L*, wire thickness *t*, wire breath *b*, and mean coil diameter *D*. If an external longitudinal force *F*_*long*_ is applied onto the end of the spring, then the corresponding longitudinal deflection *δ*_*long*_ can be described as (Wahl, 1944):

$$\delta_{\mathrm{long}}=\frac{D^{3}N_{a}}{K_{2}bt^{3}G}F_{\mathrm{long}},$$ (1)

where *N*_*a*_ is the number of active coils, *K*_2_ is a factor depending on the ratio *b*/*t*, and *G* is the spring material’s shearing modulus of rigidity. The expression in Eq. 1 can be rewritten into the common expression for a linear compression spring:

$$\delta_{\mathrm{long}}=\frac{1}{K_{\mathrm{long}}}F_{\mathrm{long}},$$ (2)

where *K*_*long*_ is commonly known as the spring’s longitudinal stiffness. It can be seen that the *free* parameters *b*, *t*, *D*, *N*_*a*_, and *G* dictate the spring’s longitudinal stiffness *K*_*long*_, and thus the amount of force to be transmitted to the MCF.

#### 2.1.2 Buckling

If a relatively large longitudinal force is exerted upon the spring, one that exceeds the spring’s critical buckling load *F*_*cr*_, then the spring would buckle and be damaged permanently. Hence it is crucial to take the buckling load into account during the design phase. The compressive *α*, flexural *β*, and shearing *γ* rigidities of a helical compression spring are needed for its computation and are given as (Wahl, 1944):

$$\alpha =\frac{K_{2}bt^{3}GL}{D^{3}N_{a}},$$ (3a)

$$\beta =\frac{2EIGI_{p}L}{N_{a}\pi D\left(GI_{p}+EI\right)},$$ (3b)

$$\gamma =\frac{8EIL}{N_{a}\pi D^{3}},$$ (3c)

Where *E* is the spring material’s modulus of elasticity, and *I* and *I*_*p*_ are the area and polar moments of inertia of the spring’s wire cross-section respectively. The spring’s critical buckling load *F*_*cr*_ is computed as (Wahl, 1944):

$$F_{cr}=\frac{\pi^{2}\beta }{LL_{1}\left(1+\frac{\pi^{2}\beta }{{L_{1}}^{2}\gamma }\right)},$$ (4)

where *L*_1_ is the critical length of the spring when *F*_*cr*_ is applied. Expression 4) can also be written in the form (Wahl, 1944):

$$F_{cr}=\frac{L-L_{1}}{L}\alpha .$$ (5)

However, at this point *L*_1_ is yet unknown. If *μ* is taken to be the ratio $\frac{L_{1}}{L}$ , then substituting *μ* into 5) and subsequently into 4) gives the following expression:

$$\mu^{3}-\mu^{2}+\frac{\mu \pi^{2}\beta }{L^{2}}\left(\frac{1}{\gamma }+\frac{1}{\alpha }\right)-\frac{\pi^{2}\beta }{L^{2}\gamma }=0,$$ (6)

which is a cubic equation that can be solved for *μ*; note that 0 < *μ* < 1. The critical buckling load *F*_*cr*_ is found by computing *L*_1_ through *μ* and substituting its value into 5).

#### 2.1.3 Lateral bending

Consider the case where the force sensor is subjected to a lateral force *F*_*lat*_ at the tip which is at a distance *L*_*f*_ from the spring’s end, as depicted in Figure 2C. The part of the force sensor along *L*_*f*_ is rigid. Hence the lateral force *F*_*lat*_ at the tip leads to a combination of a lateral force *F*_*lat,1*_ = *F*_*lat*_ and a bending moment *M*_*lat,1*_ = *F*_*lat*_ *L*_*f*_ at the spring’s end. If *s* is the arc length parameter from the spring’s base *s* = 0 to its end *s* = *L*, then the moment *M*(*s*) as a function of this arc length *s* can be given as:

$$M\left(s\right)=F_{\mathrm{lat}}\left(L+L_{f}-s\right).$$ (7)

Consequently, the parametrized curvature *κ*_*s*_(*s*) as a function of *s* going from 0 to *L* is given in the following form:

$$\kappa_{s}\left(s\right)=\frac{M\left(s\right)}{\beta }=\frac{\left(L+L_{f}-s\right)}{\beta }F_{\mathrm{lat}}.$$ (8)

The spring’s curvature *κ*_*s*_ is thus maximum at the base *s* = 0 and decreases linearly towards its end.

### 2.2 Strain sensing using FBGs

#### 2.2.1 Overall strain sensing

FBGs detect variation of strain based on the change of periodicity and refractive index of the grating. The Bragg wavelength *λ*_*B*_ is the wavelength of the light that is reflected back from the grating. The change in strain can be a result of mechanical strain *ϵ* or thermal expansion due to temperature change Δ*T*. The change in Bragg wavelength Δ*λ*_*B*_ depends on both effects and can thus be expressed as:

$$\frac{\lambda_{B}-\lambda_{B_{0}}}{\lambda_{B_{0}}}=\frac{\Delta \lambda_{B}}{\lambda_{B_{0}}}=S_{\epsilon }\Delta \epsilon +S_{T}\Delta T\;$$ (9)

where $\lambda_{B_{0}}$ is the grating’s unstrained Bragg wavelength, Δ*ϵ* is the change in mechanical strain with respect to the unstrained state, and *S*_*ϵ*_ and *S*_*T*_ are the strain and temperature sensitivity coefficients respectively.

#### 2.2.2 Decoupling mechanical and thermal strains

The force sensor embodiment comprises three FBG sets as shown in Figure 2C. As will be elaborated in Section 2.3, the central FBG is mechanically constrained between the two ends of the spring. On the other hand, the leading and trailing FBGs are free and unconstrained. This means that the middle FBG is subjected to both mechanical strain Δ*ϵ* and temperature change Δ*T*, while the leading and trailing FBGs are only subjected to temperature change Δ*T*. Hence, measuring the wavelength shifts Δ*λ*_*B*_ in the leading and trailing FBG sets allows computing Δ*T* (since Δ*ϵ* = 0) from Eq. 9, and consequently compensating for its effect in the middle FBG. This is done to isolate the mechanical strain Δ*ϵ*.

### 2.3 MCF and spring combination − longitudinal loading

#### 2.3.1 Longitudinal deflection and stiffness

**FIGURE 3:**
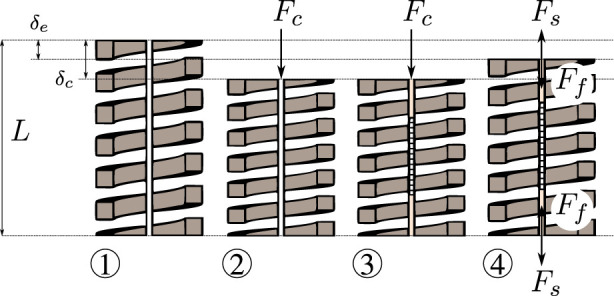
Illustration of the MCF pre-straining steps: ① the spring is fixed at the base and is initially free and unloaded, ② a longitudinal calibration force *F*_*c*_ is applied at the spring’s end, ③ the MCF is inserted into the spring and fixed at both ends with appropriate adhesives (the longitudinal calibration force *F*_*c*_ is still being applied at the spring’s end), ④ once the adhesive is fully cured, i.e., the end-points fixations are rigid, the longitudinal calibration force *F*_*c*_ is removed and the spring extends to an equilibrium position.

The MCF is rigidly fixed to both ends of the spring. In order to prevent the MCF from buckling during operation, pre-straining is applied *a priori*. The steps that are followed to apply a pre-strain on the MCF are illustrated in Figure 3 and described in the following.

① the spring is initially unloaded with its length equal to the free length *L*;
② a known longitudinal calibration force *F*_*c*_ is applied onto the spring, resulting in a deflection $\delta_{c}=\frac{F_{c}}{K_{\mathrm{long}}}$ ;
③ the MCF is introduced and rigidly fixed to the ends of the spring using adhesives (see also Figure 2C); the calibration force *F*_*c*_ is still being applied and the deflection remains *δ*_*c*_ at this point;
④ after the adhesive cures, the calibration force *F*_*c*_ is removed and the spring/fiber combination reaches a new equilibrium with the deflection of the spring being *δ*_*e*_.

At equilibrium, the internal force acting on the spring *F*_*s*_ is equal and opposite in direction to the internal force acting on the fiber *F*_*f*_. Here, the total deflection of the spring is *δ*_*e*_, while the total deflection of the fiber is *δ*_*c*_ − *δ*_*e*_. Hence, the force balance at equilibrium is:

$$K_{\mathrm{long}}\delta_{e}=K_{\mathrm{fib}}\left(\delta_{c}-\delta_{e}\right),$$ (10)

where *K*_*fib*_ is the fiber’s longitudinal stiffness. This equilibrium state is considered as the *unstrained* reference state of the fiber, i.e., where *λ*_*B*,0_ is defined and all future strain or wavelength changes are based upon. When an external longitudinal force *F*_*long*_ is applied and a new static equilibrium is reached, the following expression is obtained:

$$F_{\mathrm{long}}=K_{\mathrm{long}}\delta_{\mathrm{long}}-K_{\mathrm{fib}}\left(\delta_{c}-\delta_{\mathrm{long}}\right),$$ (11)

where *δ*_*long*_ is the spring’s longitudinal deflection due to the longitudinal force *F*_*long*_.

#### 2.3.2 Working force range

Based on the nature of the targeted application, external forces are predominantly compressive. Proper functioning of the force sensor is thus primarily related to the buckling of either the helical spring or the MCF. Buckling of the helical spring is prevented by ensuring that the magnitude of the applied external forces are $<F_{cr}$ . Similarly, buckling of the MCF is prevented by ensuring that the magnitude of the applied external forces are $<F_{c}$ . As such, both *F*_*cr*_ and *F*_*c*_ determine the force sensor’s working range. In principle, *F*_*c*_ can be as large as *F*_*cr*_. In practice, however, *F*_*c*_ is usually smaller than *F*_*cr*_. The force sensor’s working range is thus determined by *F*_*c*_ and can be customized based on application and user requirements.

#### 2.3.3 Longitudinal strain

The external longitudinal force *F*_*long*_ causing a spring deflection *δ*_*long*_ consequently corresponds to a fiber strain Δ*ϵ*_*long*_. Given that the fiber’s initial length is *L* − *δ*_*e*_, and its length after the application of the longitudinal force *F*_*long*_ is *L* − *δ*_*long*_, the fiber’s longitudinal strain Δ*ϵ*_*long*_ is thus given as:

$$\begin{array}{ll} & \Delta \epsilon_{\mathrm{long}}=\frac{\left(L-\delta_{\mathrm{long}}\right)-\left(L-\delta_{e}\right)}{L-\delta_{e}},\\ & \;\;=\frac{\delta_{e}-\delta_{\mathrm{long}}}{L-\delta_{e}}.\; \end{array}$$ (12)

Considering that *L* ≫ *δ*_*e*_, hence *L* ≈ *L* − *δ*_*e*_, expression (12) can be simplified to:

$$\Delta \epsilon_{\mathrm{long}}=\frac{\delta_{e}-\delta_{\mathrm{long}}}{L}.$$ (13)

Substituting (13) into 9) gives the longitudinal deflection *δ*_*long*_ due to the applied longitudinal force *F*_*long*_ from a measured wavelength shift Δ*λ*_*B*_:

$$\delta_{\mathrm{long}}=\delta_{e}-\frac{L}{S_{\epsilon }}\left(\frac{\Delta \lambda_{B}}{\lambda_{B_{0}}}-S_{T}\Delta T\right).$$ (14)

By combining expressions (10), (11) and (14), and solving for *F*_*long*_, the following expression is obtained:

$$F_{\mathrm{long}}=\left(K_{\mathrm{long}}+K_{\mathrm{fib}}\right)\frac{L}{S_{\epsilon }}\left(S_{T}\Delta T-\frac{\Delta \lambda_{B}}{\lambda_{B_{0}}}\right).$$ (15)

If we consider that the temperature is constant or compensated, then expression (15) can be simplified to obtain the following longitudinal force *F*_*long*_ magnitude:

$$F_{\mathrm{long}}=\frac{\left(K_{\mathrm{long}}+K_{\mathrm{fib}}\right)L}{S_{\epsilon }\lambda_{B_{0}}}\Delta \lambda_{B}.$$ (16)

The magnitude of the externally applied longitudinal force *F*_*long*_ could thus be obtained *via* the linear relationship in Eq. 16 by measuring the wavelength shift Δ*λ*_*B*_.

#### 2.3.4 Longitudinal force resolution

The wavelength shift Δ*λ*_*B*_ within the MCF is usually measured using an optical interrogator. The interrogator, such as one based on wavelength division multiplexing (WDM), can measure wavelength shifts to a given resolution. The resolution is usually related to the interrogator’s hardware and the accuracy of the subsequent wavelengths peak detection algorithm. Accordingly, Λ_*min*_ is defined as the minimum detectable wavelength shift. The longitudinal force resolution Ω_*long*_ can thus be obtained by solving (16) and substituting Δ*λ*_*B*_ with Λ_*min*_ such that:

$$\Omega_{\mathrm{long}}=\frac{\left(K_{\mathrm{long}}+K_{\mathrm{fib}}\right)L}{S_{\epsilon }\lambda_{B_{0}}}\Lambda_{\min}.$$ (17)

### 2.4 MCF and spring combination − lateral loading

#### 2.4.1 Effective rigidity and curvature

Figure 2C illustrates how the fiber is fixed with respect to the helical spring. Ideally, the fiber is concentric and collinear with respect to the spring’s central longitudinal axis. The fiber is rigidly fixed to the ends of the spring using adhesives. Assuming equal curvature and relatively *small* deflections, the fiber and spring combination can be treated as a composite material having an effective flexural rigidity *β*_*eff*_ as (Denavit et al., 2018):

$$\beta_{\mathrm{eff}}=E_{\mathrm{fib}}I_{\mathrm{fib}}+\beta ,$$ (18)

where *E*_*fib*_ and *I*_*fib*_ are the fiber’s modulus of elasticity and cross-sectional area moment of inertia respectively. Expression 8) can then be updated to include this composition for the effective curvature *κ*_*eff*_:

$$\kappa_{\mathrm{eff}}\left(s\right)=\frac{\left(L+L_{f}-s\right)}{\beta_{\mathrm{eff}}}F_{\mathrm{lat}}.$$ (19)

The position of the middle FBG, i.e., the FBG constrained between both ends of the spring, can be adjusted along the longitudinal axis of the spring before rigidly fixing it. This will define the arc length *s* at which wavelength and strain measurements are evaluated. If we consider the location of the FBG to be at *s* = *L*_*FBG*_, i.e., the distance to the FBG’s center along its length, and take *L*_*eff*_ = *L* + *L*_*f*_ − *L*_*FBG*_ and *κ*_*eff*_ ≡ *κ*_*eff*_ (*L*_*FBG*_), then the magnitude of the applied lateral force *F*_*lat*_ can be given as:

$$F_{\mathrm{lat}}=\frac{\beta_{\mathrm{eff}}}{L_{\mathrm{eff}}}\kappa_{\mathrm{eff}}.$$ (20)

Expression (20) illustrates the linear relationship between the lateral force magnitude *F*_*lat*_ and effective curvature *κ*_*eff*_.

#### 2.4.2 Lateral force resolution

**FIGURE 4:**
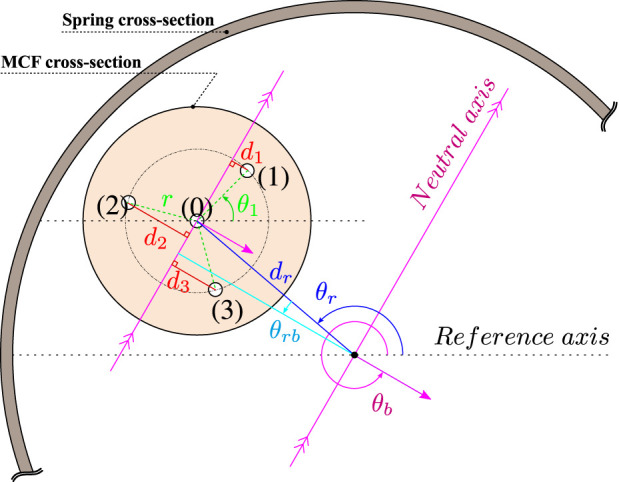
Cross-sectional view of the MCF and spring combination. The MCF’s center is at a radial offset *d*_*r*_ from the spring’s center and at an angular offset *θ*_*r*_ with respect to a reference axis. *θ*_*b*_ is the angle of the bending plane vector with respect to the reference axis and *θ*_1_ is the angle between the center of the first outer core and the reference axis. *r* is the radial distance from an outer core to the central core, and *d*_*i*_ for $i\in \left[1,2,3\right]$ is the perpendicular distance between the outer cores and the neutral axis crossing the central core. *θ*_*rb*_ is the angle between the bending plane vector *θ*_*b*_ − *π* and *θ*_*r*_.

With reference to Figure 4, considering a MCF configuration with one central core and three equally distributed outer cores along a circumference with a radial distance *r* from the center, the relationship between measured strain *ϵ* and effective curvature *κ*_*eff*_ is (Moore and Rogge, 2012):

$$\kappa_{\mathrm{eff}}=\frac{-\epsilon_{i}}{r\text{cos}\left(\theta_{b}-\frac{3\pi }{2}-\theta_{i}\right)},$$ (21)

where $i\in \left[1,2,3\right]$ represents the *i*th outer core, *θ*_*b*_ is the angle of the bending plane normal vector, $\theta_{i}=\theta_{1}+\frac{2\pi \left(i-1\right)}{3}$ , and *θ*_1_ is the angle between the center of the first outer core and a reference axis. The reference axis can be arbitrarily chosen but usually coincides with an externally identifiable geometric landmark. The magnitude of the measured effective curvature *κ*_*eff*_ can be found as follows (Moore and Rogge, 2012):

$$\kappa_{\mathrm{eff}}=\frac{2}{3r}\sqrt{{\left(\sum_{i=1}^{3}\epsilon_{i}\text{cos}\theta_{i}\right)}^{2}+{\left(\sum_{i=1}^{3}\epsilon_{i}\text{sin}\theta_{i}\right)}^{2}}.$$ (22)

The angle of the bending plane normal vector *θ*_*b*_ can be obtained from the apparent curvature vector ***κ***_*app*_ as given in (Moore and Rogge, 2012), and is used to divide the total curvature *κ*_*eff*_ into its orthogonal principal components. By replacing the expression for *κ*_*eff*_ from Eq. 22 into Eq. 20, the following is obtained:

$$F_{\mathrm{lat}}=\frac{2\beta_{\mathrm{eff}}}{3rL_{\mathrm{eff}}}\sqrt{{\left(\sum_{i=1}^{3}\epsilon_{i}\text{cos}\theta_{i}\right)}^{2}+{\left(\sum_{i=1}^{3}\epsilon_{i}\text{sin}\theta_{i}\right)}^{2}}.$$ (23)

Without loss of generality, a simplified geometrical case can be analysed to derive an overall estimate of the lateral force resolution Ω_*lat*_. Here, the reference axis is considered to coincide with the first outer core, i.e., *θ*_1_ = 0, and the bending plane coincides with the reference axis, i.e., $\theta_{b}=\frac{3\pi }{2}$ . Due to symmetry, the sum of the outer core strains must be equal to zero, $\sum_{i=1}^{3}\epsilon_{i}=0$ . Given that the measured strain in the first outer core will be zero (since it falls along the neutral axis), the strains in the second and third outer cores will be equal in magnitude but opposite in direction such that *ϵ*_2_ = −*ϵ*_3_. The resulting relationship between the lateral force *F*_*lat*_ and measured strain *ϵ*_2_ based on Eq. 23 is found to be:

$$F_{\mathrm{lat}}=\frac{2\beta_{\mathrm{eff}}}{\sqrt{3}rL_{\mathrm{eff}}}\epsilon_{2}.$$ (24)

Similar to the longitudinal case, by substituting Δ*λ*_*B*_ with Λ_*min*_ into 9), considering that the temperature is constant or compensated, and taking *ϵ*_2_ = Δ*ϵ*, the following expression is found from Eq. 24 for the lateral force resolution Ω_*lat*_:

$$\Omega_{\mathrm{lat}}=\frac{2\beta_{\mathrm{eff}}}{\sqrt{3}rL_{\mathrm{eff}}S_{\epsilon }\lambda_{B,0}}\Lambda_{\min}.$$ (25)

The longitudinal and lateral force resolutions, Ω_*long*_ and Ω_*lat*_, could thus be customized by dimensioning the various sensor’s properties and geometry. Note that the force resolutions are not strictly decoupled and thus modification of one may have an effect on the other.

## 3 Decoupling longitudinal and lateral forces

### 3.1 Total strain

The MCF is ideally embedded coaxially with the spring’s longitudinal axis. However, it is a challenge to practically guarantee this. It therefore becomes necessary to compensate for any of the adverse effects that result from MCF and spring non-coaxiality. Figure 4 provides an illustrative schematic of the cross-sectional view of a general MCF and spring combination. The MCF’s center is at a certain radial offset *d*_*r*_ from the spring’s longitudinal axis and at an angular offset *θ*_*r*_ from the reference axis. The perpendicular distance between the line parallel to the neutral axis crossing the MCF’s central core and the spring’s longitudinal axis is given by *d*_*r*_ cos *θ*_*rb*_, where *θ*_*rb*_ = *θ*_*b*_ − *π* − *θ*_*r*_. The total measured strain in each of the MCF’s cores when both a longitudinal force *F*_*long*_ and a lateral force *F*_*lat*_ are applied can thus be given as follows:

$$\epsilon_{0}=\epsilon_{\mathrm{long}}+\epsilon_{\Delta T}+d_{r}\text{cos}\theta_{rb}\kappa_{\mathrm{eff}},\;\;\;\;\;\;\;\;\;\;\;\;\;\;\;\;\;\;$$ (26a)

$$\epsilon_{i}=\epsilon_{\mathrm{long}}+\epsilon_{\Delta T}+d_{r}\text{cos}\theta_{rb}\kappa_{\mathrm{eff}}-r\text{cos}\left(\theta_{b}-\frac{3\pi }{2}-\theta_{i}\right)\kappa_{\mathrm{eff}},$$ (26b)

Where *ϵ*_0_ is the strain in the central core, *ϵ*_*i*_ is the strain in the *i*th outer core for $i\in \left[1,2,3\right]$ , and *ϵ*_Δ*T*_ is the strain due to temperature change. As can be seen from (26), the common mode amongst all cores is measured with the central core and contains the effects from longitudinal strain *ϵ*_*long*_, temperature change *ϵ*_Δ*T*_, and offset from the neutral axis *d*_*r*_ cos *θ*_*rb*_ *κ*_*eff*_.

### 3.2 Decoupling of strains

Without loss of generality, the reference axis will be chosen to be parallel with the line intersecting the MCF’s central and first outer cores such that *θ*_1_ = 0. This is done to obtain a straightforward solution and avoid extra calibration to find *θ*_1_. The assignment is possible since the choice of the direction of the reference axis can be arbitrarily defined. Accordingly, the remaining unknowns in (26) are the geometrical offsets *d*_*r*_ and *θ*_*r*_. These parameters are found through calibration and least-squares fitting (as will be seen later in Section 4). Note that the curvature magnitude *κ*_*eff*_, as defined in Eq. 22, and the direction of the bending plane normal vector *θ*_*b*_ are independent from the common mode. This is because their computation relies on the differences in strain between the outer cores and not on their absolute magnitude. Hence both *κ*_*eff*_ and *θ*_*b*_ can be independently computed from the outer core strains *ϵ*_*i*_. This can then be used to compute the lateral force *F*_*lat*_ *via* Eq. 20. Note that the lateral force can be decomposed into its orthogonal principal components using the bend angle *θ*_*b*_. As previously elaborated, the force sensor embodiment allows for the compensation of temperature changes by employing two extra unstrained FBGs. Since the FBGs are unstrained, i.e., Δ*ϵ* = 0, their measured wavelength shifts Δ*λ*_*B*_ are used to compute temperature changes Δ*T*. The longitudinal strain *ϵ*_*long*_ could thus be computed from Eq. 26a, converted to a longitudinal wavelength shift Δ*λ*_*B*,*long*_ using (9), and consequently used to compute the longitudinal force *F*_*long*_ from Eq. 16. In this manner, both *F*_*long*_ and *F*_*lat*_ can be computed separately and independently.

## 4 Sensor development and calibration

### 4.1 MCF pre-straining

**FIGURE 5:**
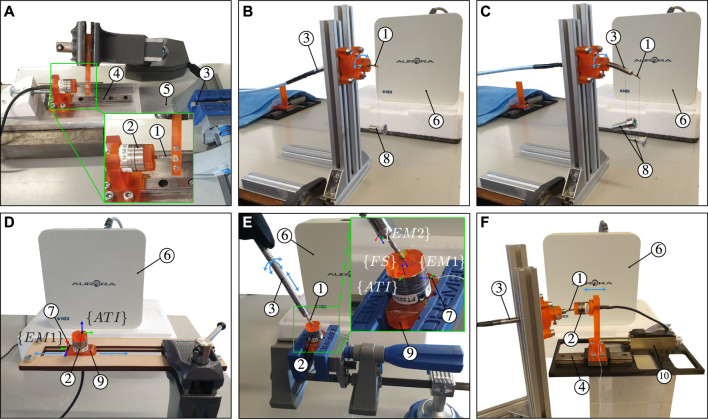
Illustration of the calibration and experimental setups for **(A)** MCF pre-straining through spring initial compression **(B)** identification of *d*_*r*_ and *θ*_*r*_ **(C)** identification of the angle between the *x* − axis of EM two and the first outer core of the MCF **(D)** identification of the angle between the *x* −axis of EM one and the *y* −axis of the ATI force sensor **(E)** three-dimensional catheter tip force sensor calibration, and **(F)** dynamic force characterization. The calibration and experimental setups contain the following elements: ① catheter tip force sensor, ② ATI Nano17 force sensor, ③ bendable and actuatable catheter, ④ linear guide, ⑤ MCF, ⑥ EM field generator, ⑦ EM two sensor, ⑧ hanging weights, ⑨ 3D printed rigid base, and ⑩ linear servomotor.

Figure 5A illustrates the experimental setup used for MCF pre-straining. The force sensor body comprising the flexural spring is held firmly using a mechanical vice. A commercial six DoF Nano17 force/torque sensor (ATI Industrial Automation, Apex, NC, United States) is used to measure and apply the compressive calibration force *F*_*c*_. The ATI force sensor is mounted onto a sliding block and a linear guide. A screw at the backside of the ATI force sensor is used for incremental displacement and control of the contact force. Once the desired calibration force *F*_*c*_ is applied, the screw is fixed in place and adhesives are applied onto the MCF and spring combination. The ATI force sensor is moved back and released once the two-point adhesive curing is complete.

### 4.2 Computing offsets *d*_*r*_ and *θ*_*r*_

Consider a configuration *j*, where the force sensor is clamped horizontally with a predominantly lateral force being applied at the tip in the direction of gravity, as shown in Figure 5B. Consider the exact same configuration *j* + 1 where the force sensor is rotated by an incremental amount about its longitudinal axis. From Eq. 26a, the strain relationship in the central core for those two configurations would be as follows:

$$\epsilon_{0,j}=\epsilon_{\mathrm{long,j}}+\epsilon_{\Delta T,j}+d_{r}\text{cos}\theta_{rb,j}\kappa_{\mathrm{eff,j}},$$ (27a)

$$\epsilon_{0,j+1}=\epsilon_{\mathrm{long,j+1}}+\epsilon_{\Delta T,j+1}+d_{r}\text{cos}\theta_{rb,j+1}\kappa_{\mathrm{eff,j+1}}.$$ (27b)

Given the axisymmetry of the spring and force sensor body, the curvature *κ*_*eff*_ should remain approximately constant between the two configurations such that *κ*_*eff*_ = *κ*_*eff,j*_ = *κ*_*eff,j+1*_. Furthermore, the corresponding longitudinal strain in both configurations should also remain approximately the same, *ϵ*_*long,j*_ = *ϵ*_*long,j+1*_. Finally, if the two configurations are achieved within a short period of time, or the environmental temperature is kept constant, then *ϵ*_Δ*T*,*j*_ = *ϵ*_Δ*T*,*j*+1_. Comparing both configurations would thus yield:

$$\epsilon_{0,j+1}-\epsilon_{0,j}=d_{r}\kappa_{\mathrm{eff}}\left(\text{cos}\theta_{rb,j+1}-\text{cos}\theta_{rb,j}\right).$$ (28)

Considering that there are *N* consecutive configurations differing by an incremental rotation about the force sensor’s longitudinal axis, then a vector **Ψ** with *N* − 1 elements can be constructed such that:

$$\Psi \left(j\right)=\epsilon_{0,j+1}-\epsilon_{0,j}-d_{r}\kappa_{\mathrm{eff}}\left(\text{cos}\theta_{rb,j+1}-\text{cos}\theta_{rb,j}\right),$$ (29)

where **Ψ**(*j*) is the *j*th element of **Ψ**. The geometrical offsets *d*_*r*_ and *θ*_*r*_ can be obtained through least-squares minimization of **Ψ**, i.e., $\min\left(\sum_{j=1}^{N-1}{\Psi \left(j\right)}^{2}\right)$ . Note that *d*_*r*_ is constrained between $\left[0,d_{r,\max}\right]$ , where *d*_*r*, *max*_ is the maximum possible central offset of the MCF within the spring, and *θ*_*r*_ is constrained between $\left[0,2\pi \right]$ .

### 4.3 Calibration matrix

Following from the relationships given in Eq. 16 and Eq. 20, it is clear that the longitudinal *F*_*long*_ and lateral *F*_*lat*_ forces vary linearly with the measurements Δ*λ*_*B*,*long*_ and *κ*_*eff*_, respectively. A coordinate frame is assigned to the force sensor such that the *xy* − axes fall on a plane with a normal in the direction of the spring’s longitudinal axis, and the *z* − axis points in the direction of that normal, from the base to the tip (see Figure 5E). Considering that *F*_*x*_ = *F*_*lat,x*_, *F*_*y*_ = *F*_*lat,y*_, and *F*_*z*_ = *F*_*long*_, then the overall relationship between the output force and the input measurements is given as:

F=CΘ, (30a)

$$\left[\begin{array}{c} F_{x}\\ F_{y}\\ F_{z} \end{array}\right]=\left[\begin{array}{ccc} c_{11} & c_{12} & c_{13}\\ c_{21} & c_{22} & c_{23}\\ c_{31} & c_{32} & c_{33} \end{array}\right]\times \left[\begin{array}{c} \kappa_{\mathrm{eff}}\;\text{cos}\left(\theta_{b}\right)\\ \kappa_{\mathrm{eff}}\;\text{sin}\left(\theta_{b}\right)\\ \Delta \lambda_{B,long} \end{array}\right],$$ (30b)

Where $F\;=\;\left[F_{x}\right.$ , *F*_*y*_, ${\left.F_{z}\right]}^{T}$ , $\Theta \;=\;\left[\kappa_{\mathrm{eff}}\right.\text{cos}\left(\theta_{b}\right)$ , $\kappa_{\mathrm{eff}}\;\text{sin}\left(\theta_{b}\right)$ , ${\left.\Delta \lambda_{B,long}\right]}^{T}$ , and ***C*** is the calibration matrix. The calibration matrix ***C*** is found from experimental calibration data for ***F*** and **Θ** and computed as ***C*** = ***F*Θ** ^**+**^, where ^+^ represents the matrix pseudo-inverse.

### 4.4 Force calibration setup

Calibration of the force sensor requires 3D ground truth force data, obtained here by utilizing the commercial Nano17 force/torque sensor. A transformation of the measured 3D force from the Nano17 force sensor’s coordinate frame to the local frame of the developed force sensor is required however. This is achieved by using the setup shown in Figure 5E. Two EM sensors are employed here to establish the coordinate frame transformation between the Nano17 ground truth and catheter tip force sensors. The first EM is attached to a rigid base which in turn is attached to the Nano17 force sensor. The second EM is embedded within the notched tube catheter which in turn houses the developed force sensor. The transformations between the Nano17 force sensor and EM 1, and the developed force sensor and EM 2, are thus known and constant. The method to obtain these transformations is elaborated next.

#### 4.4.1 Transformation from Nano17 to EM 1

The rigid base shown in Figure 5E is constructed such that the *z* − axis of the EM sensor is parallel with the *x* − axis of the Nano17 force sensor. This means that the *xy* plane of the EM sensor is parallel to the *yz* plane of the Nano17 force sensor. The angle between the *x* − axis of the EM sensor with respect to the *xy* plane of the Nano17 force sensor *θ*_*x*1_, is required to fully define the rotational transformation from the Nano17 force sensor frame {*ATI*} to the EM sensor frame {*EM*1}. The rigid base has flat surfaces at opposite sides parallel to the *xy* plane of EM 1. Accordingly, the rigid base is placed in a slot constraining its movement exclusively along the *y* − axis of the Nano17 force sensor (see Figure 5D). The rigid base is moved back and forth within this slot while simultaneously measuring EM one position data. This generates a series of three-dimensional points through which a straight line can be fitted. Angle *θ*_*x*1_ is then found by computing the angle between the *x* − axis of EM one and the fitted line.

#### 4.4.2 Transformation from EM two to force sensor

The notched tube catheter shown in Figure 5E is constructed such that the *z* − axis of the EM sensor is parallel with the local *z* − axis of the developed force sensor. Accordingly, the notched tube catheter and force sensor assembly are clamped horizontally. A weight is attached to the notched tube catheter and a lighter weight is attached to the force sensor (see Figure 5C). Both the notched tube catheter and the force sensor would bend in the same direction due to gravity. A vector can then be constructed from the position of the EM when no weight is applied to the position of the EM when the weights are applied. This vector is projected onto the *xy* plane of the EM sensor and used to compute the angle between this vector and the *x* − axis of the EM sensor *θ*_*x*2_. The process is repeated for different orientations, i.e., rotation about the longitudinal axis, to obtain better approximation of *θ*_*x*2_. The *x* − axis of EM two and the line between the central and first outer core are aligned by rotating the EM frame {*EM*2} about its *z* − axis by *θ*_*b*_ − 2*π* − *θ*_*x*2_.

#### 4.4.3 Total transformation

The 3D ground truth forces measured by the Nano17 force sensor are transformed to the force sensor frame {*FS*} by following the consecutive transformation order {*ATI*} → {*EM*1} → {*EM*2} → {*FS*}.

#### 4.4.4 Calibration procedure

The experimental setup to carry out the force calibration procedure is illustrated in Figure 5E. As previously stated, the force sensor is integrated within the body of an in-house catheter. The catheter is held, by hand, at a proximal point close to the force sensor. The force sensor tip is then directed towards the surface of the ground truth ATI force sensor and made to establish contact. Application of three-dimensional forces with varying magnitudes upon the force sensor’s tip is achieved by simply changing the orientation of the force sensor with respect to the ATI sensor and changing the contact magnitude (by manually pressing or releasing). Display of the three-dimensional forces is provided in real-time to aid with the application of diversified forces and to prevent applying excessive forces.

### 4.5 Temperature compensation

The temperature compensation capability of the catheter tip force sensor plays an important role in its overall applicability and performance. This is especially the case given that it is meant to be operated in an environment with continuous thermal variation. The force sensor’s design allows for this temperature compensation capability by employing two extra free FBG sets placed at either sides of the mechanically strained central FBG. Considering a state with no mechanical strain, i.e., Δ*ϵ* = 0, temperature change Δ*T* follows a direct linear relationship with wavelength shift Δ*λ*_*B*_, as presented in Eq. 9. The goal is thus to characterize this linear relationship between Δ*T* and Δ*λ*_*B*_ for the three FBG sets. Accordingly, when a temperature change occurs, the two free FBG sets are employed to compute the temperature change Δ*T* from their wavelength shifts Δ*λ*_*B*_. This is then used to compensate for the temperature effect in the central FBG and isolate the mechanical strain Δ*ϵ*. To characterize the relationships between Δ*T* and Δ*λ*_*B*_ for the three FBG sets, the setup shown in Figure 5B is placed into a temperature controlled chamber. The temperature is then cyclically increased and decreased for a number of cycles. Temperature and wavelength shift data are gathered during this process. The process is carried out twice: *1)* when the force sensor is free and unloaded, i.e., for the purpose of characterization, and *2)* when a load hangs from the force sensor, i.e., for the purpose of validating the sensor’s temperature compensation capability.

### 4.6 Dynamic force behaviour

Catheter ablation procedures are commonly performed on beating heart tissue. The force sensor must thus be able to cope with the dynamic heart environment and provide accurate contact force estimates for the complete range of heart motion frequencies. Accordingly, the experimental setup depicted in Figure 5F is constructed to characterize the dynamic performance of the force sensor. The Nano17 force sensor is mounted onto a linear guide and rail which is in turn connected to a LM2070 linear servomotor (Faulhaber, Schonaich, Germany). The servomotor can be controlled with micrometer accuracy to apply desired forces on the catheter tip force sensor. Sinusoidal force profiles with varying frequencies were applied to impose a varying loading on the force sensor. Ground truth and estimated forces are consequently used for dynamic performance characterization.

## 5 Results and discussion

This section presents the obtained results in relation to the force sensor’s calibration and corresponding force sensor’s performance with respect to various aspects. These include: 1) overall sensor properties and theoretical longitudinal and lateral force resolutions, 2) effect and evaluation of MCF geometrical offsets *d*_*r*_ and *θ*_*r*_, 3) evaluation of transformation parameters *θ*_*x*1_ and *θ*_*x*2_ needed for the force calibration, 4) experimental force calibration matrix and longitudinal and lateral force resolutions, 5) resulting force sensor accuracy and obtained measurement errors, 6) sensor’s temperature compensation capability, and 7) force sensor’s behaviour under dynamic loading and overall repeatability.

### 5.1 Sensor parameters

The force sensor prototype was dimensioned based on a set of geometrical and manufacturing constraints. These were mainly concerning the sensor’s outer diameter, material, helical pitch, and total length. The resulting sensor parameter values are summarized in Table 1. Accordingly, the developed prototype comprises a total length of 16.3 mm from base to tip and an outer diameter of 2.2 mm. The MCF has the following properties: *r* = 37.5 ⋅ 10^–6^ m, *S*_*ϵ*_ = 0.777, $\lambda_{B_{0}}=1582$ nm, one central core, three equally spaced outer cores, a FBG center-to-center distance of 6 mm, a FBG length of 2 mm, and a longitudinal stiffness of *K*_*fib*_ = 1.91 ⋅ 10^5^ N/m. Wavelength shifts were measured by a FBG-Scan 908 EP wavelength division multiplexing (WDM) based interrogator (FBGS International NV, Geel, Belgium) with a minimum detectable wavelength shift of Λ_*min*_ = 0.01 nm. The resulting theoretical longitudinal and lateral force resolutions, Ω_*long*_ and Ω_*lat*_, are thus found to be 8.59 ⋅ 10^–3^ N and 8.35 ⋅ 10^–4^ N respectively. Furthermore, according to 5) and 6), the spring’s critical buckling load *F*_*cr*_ is found to be around 16 N. This value is far greater than the expected forces subjected to the catheter which can reach 4.5 N (Srimathveeravalli et al., 2010). Note, however, that the force sensor was constructed to demonstrate its performance in the clinically determined force ablation range ≈0.1−0.4 N. As such, the applied pre-strain calibration force *F*_*c*_ was chosen to be 0.8 N, which also determines the force sensor’s working range.

**TABLE 1:** Sensor parameter definitions and quantitative values.

Symbol	Definition	Value	Unit
*L*	Spring free length	4.50	mm
*t*	Spring wire thickness	0.37	mm
*b*	Spring wire breadth	0.35	mm
*D*	Spring mean coil diameter	1.85	mm
*N*_*a*_	Spring number of active coils	6	−
*K*_2_	Spring factor	1.22	−
*E*	Spring material modulus of elasticity	193	GPa
*G*	Spring material modulus of rigidity	77	GPa
*L*_*f*_	Sensor rigid section length	6.80	mm
*L*_*FBG*_	Location of FBG along spring arc length	2.00	mm

### 5.2 Identification of *d*_*r*_ and *θ*_*r*_

**FIGURE 6:**
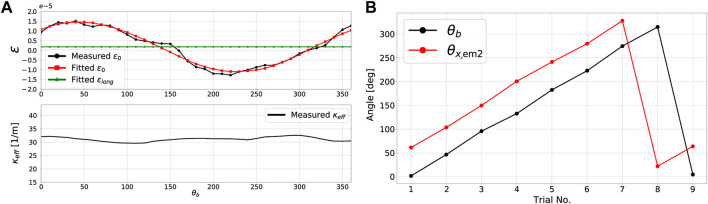
Catheter tip force sensor calibration results: **(A)** central core strain and curvature profiles during the identification of *d*_*r*_ and *θ*_*r*_, and **(B)** *θ*_*b*_ and *θ*_*x*2_ profiles for different force sensor rotational configurations about its longitudinal axis.

Figure 6A shows the results of the experimental procedure carried out for the identification of *d*_*r*_ and *θ*_*r*_. The force sensor was rotated about its longitudinal axis within a range of 0–360° at increments of approximately 10°. The hanging weight and other environmental conditions were kept constant throughout the experiment. As expected, the effective curvature *κ*_*eff*_ remained almost constant for all angular configurations, varying with less than 10% of its total magnitude. Figure 6A also clearly shows that the measured strain in the central core *ϵ*_0_ does not remain constant but rather varies sinusoidally with the orientation of the load. As previously shown in (26a), the strain in the central core *ϵ*_0_ is a combination of longitudinal strain *ϵ*_*long*_ and bending due to a geometric offset from the center *d*_*r*_ cos *θ*_*rb*_ *κ*_*eff*_. The values for *ϵ*_*long*_, *d*_*r*_ and *θ*_*r*_ were identified through least squares fitting using the data for *ϵ*_0_ and found to be 2 ⋅ 10^–6^, 4.1 ⋅ 10^–7^ m, and 3.97 rad respectively.

### 5.3 Identification of angles *θ*_*x*1_ and *θ*_*x*2_

The procedure for the identification of EM angles *θ*_*x*1_ and *θ*_*x*2_ was straightforward as previously outlined in Section 4.4. A 3D line was fitted from position data of EM one during its motion along the constrained 1D slot shown in Figure 5D. Angle *θ*_*x*1_, which is the angle between the *x* − axis of EM one and this 3D line, was found to be *θ*_*x*1_ = 3.96 rad Figure 6B shows the profiles of the bend angle *θ*_*b*_ and the angle of the *x* − axis of EM 2 with respect to the direction of gravity, *θ*_*x*,*em*2_, for different catheter and force sensor rotational configurations. As shown in Figure 6B, it is clear that the difference between the two angles is constant for the different rotational configurations. This difference is the definition of *θ*_*x*2_ and was found to be around 59.6°.

### 5.4 Force calibration and resolution

The force calibration procedure involved gathering several data including: *1)* FBG wavelength shifts, *2)* EM sensor poses, and *3)* 3D ground truth forces. Data coming from the different sources were synchronized in time to obtain a data sampling rate of 40 Hz. Note that this limitation in sampling frequency is due to the commercial EM system. The MCF wavelength shifts can be in principle measured at around 250 Hz. The wavelength shifts were used to compute the input measurement vector **Θ**, while the 3D ground truth forces and EM poses were used to compute the force vector ***F***. Variation of the force vector’s *xyz* components was obtained by modifying the pose and contact force of the catheter tip force sensor with respect to the Nano17 force sensor. The obtained force ranges for the *xyz* components of ***F*** were 0.40 N, 0.35 N, and 0.51 N respectively. The obtained standard deviation of the applied *xyz* force components of ***F*** were 0.072 N, 0.071 N, and 0.11 N respectively. The calibration data set contained data for a total duration of around 760 s. The calibration matrix ***C*** = ***F*Θ** ^**+**^ was found to be:

$$C=\;\left[\begin{array}{ccc} -0.003556 & 0.000079 & -0.003682\\ 0.000061 & -0.003932 & -0.008789\\ 0.000003 & 0.000369 & -0.749846 \end{array}\right].$$

The decoupling of lateral and longitudinal forces with respect to their respective measurements can be clearly seen from the calibration matrix ***C***, but also from the cross-correlation matrix ***R***_***cc***_:

$$R_{cc}=\;\left[\begin{array}{ccc} -0.992596 & 0.262518 & -0.004549\\ 0.255637 & -0.985295 & 0.198597\\ -0.006603 & 0.222581 & -0.986881 \end{array}\right].$$

A measurement vector **Θ**_***min***_ based on the minimum wavelength shift Λ_*min*_ can be constructed such that $\Theta_{\min}\;=\;\left[\kappa_{\mathrm{eff,min}}\right.$ , *κ*_*eff,min*_, ${\left.\Lambda_{\min}\right]}^{T}$ , where *κ*_*eff,min*_ is found through replacing Λ_*min*_ into 9) and consequently into (21). The actual force resolution could thus be found from ***C*Θ**_***min***_ yielding longitudinal and lateral force resolutions of 7.42 ⋅ 10^–3^ N and 8.07 ⋅ 10^–4^ N, respectively. It can be seen that the actual and theoretical force resolutions are highly comparable, both for the longitudinal and lateral cases. This validates the proposed theoretical sensor design approach and allows for sensor dimensioning based on predetermined performance criteria. Note that in principle, the longitudinal and lateral force resolutions can be made equal by dimensioning the sensor parameters to be constrained such that Ω_*long*_ = Ω_*lat*_, i.e., equating expressions (17) and (25). This however, may sometimes not be possible due to additional geometric and manufacturing constraints, as was the case in this work.

### 5.5 Performance validation

**FIGURE 7:**
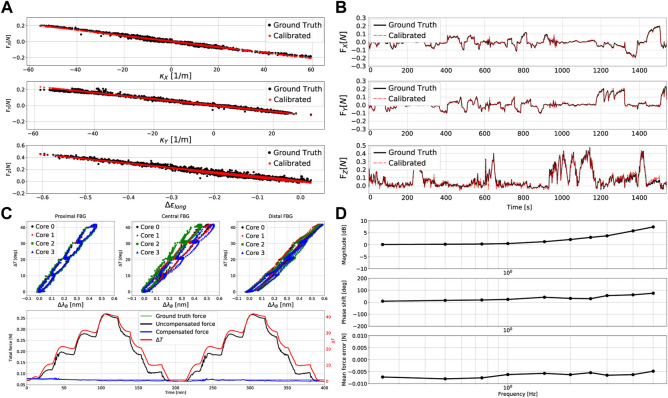
Catheter tip force sensor calibration results: **(A)** relationships between the 3D measured and calibrated forces *versus* input measurements *κ*_*x*_, *κ*_*y*_ and Δ*ϵ*_*long*_, **(B)** comparison of the ground truth and estimated 3D forces for different force configurations across time, **(C)** relationships between temperature change and wavelength shift for the three FBG sets, and comparison of temperature compensated and temperature uncompensated force estimations, **(D)** dynamic frequency-based force estimation bode plot and mean force error *versus* frequency.

Besides the previously outlined calibration dataset, a separate dataset was measured to validate the sensor’s performance. The data gathering methodology was identical to what has been previously outlined. Synchronized data were again measured at a sampling rate of 40 Hz for an increased total duration of around 1540 s. Figure 7A shows the results of this procedure employing the previously obtained calibration matrix ***C***. Two key observations can be made: *1)* the variation of force output follows a clear linear relationship with measurement input, and *2)* hysteresis is negligible as the data exhibits a minimum *R* ^2^ value of 0.968 between force output and measurement input. Furthermore, Figure 7B shows the temporal evolution of ground truth and calibrated forces during the data gathering procedure. Table 2 provides a summary of the corresponding force errors between the ground truth and calibrated forces. Longitudinal force estimations remain primarily within a ±0.018 N error range, while lateral force estimations remain primarily within a ±0.010 N error range. The results show that the force estimation accuracy of the developed sensor is either comparable, or superior, to other state of the art force sensors.

**TABLE 2:** Validated sensor performance (STD. = standard deviation). Percentages are with respect to the maximum applied forces.

Force component	Max. Applied force [mN]	Mean error [mN]	STD. of error [mN]	Maximum error [mN]
*F*_*x*_	210	−0.28 (0.13%)	8.27 (3.94%)	69.8 (33.3%)
*F*_*y*_	230	−0.96 (0.42%)	9.91 (4.31%)	57.3 (24.9%)
*F*_*z*_	470	−0.72 (0.15%)	17.9 (3.80%)	175.6 (37.4%)

### 5.6 Temperature compensation

Temperature characterization experiments involved varying the temperature from around 20*°*C–60*°*C and back with 10*°*C steps for two complete cycles. This was initially done with the sensor being free and unloaded in order to characterize the temperature and wavelength shift relationship. The same procedure was then carried out again with a load hanging from the force sensor. Note that the temperature variation was limited to 60*°*C to protect the catheter and its constituents from permanent damage. The experiment was performed to prove the force sensor’s temperature compensation capability, and is thus expected to operate similarly for higher temperature ranges. The results of these temperature characterization experiments are shown in Figure 7C (top three plots). As predicted, the relationship between temperature change Δ*T* and wavelength shift Δ*λ*_*B*_ is fairly linear for all FBG sets. The minimum *R* ^2^ value amongst these linear relationships was found to be 0.968. Figure 7C also depicts the evolution of temperature across time and illustrates the sensor’s temperature uncompensated and compensated force estimations. It is clear that without temperature compensation, the estimated force drifts far away from the ground truth, resulting in poor sensor performance. On the other hand, incorporating temperature compensation allows for eliminating the effect of temperature on the output force estimation. Temperature compensation maintained force estimation errors within a maximum of ±0.008 N. Hence, it becomes evident that the overall sensor performance is maintained over the wide range of temperature variations.

### 5.7 Dynamic force behaviour and repeatability

Considering that the force sensor is expected to operate within a dynamic beating heart environment, characterizing its behaviour under such conditions is crucial to asses the force sensor’s performance. Although average human heart motion frequencies range between 1–2 Hz (Chang et al., 2018), the force sensor was subjected to sinusoidal force profiles with frequencies from 0.25 Hz up to 5 Hz to explore its dynamic limits. The results of these dynamic characterization experiments are shown in the bode and error plots of Figure 7D. Sensor gains and phase shifts varied from 0.1–7.4 dB and 8.5–75.1° respectively, for the previously outlined range of frequencies. The mean force error magnitude however, remained almost constant at around 0.006 N. The sensor gain remained under 3 dB for frequencies ≤2.5 Hz, with a phase shift of 29° at that point. This means that the system’s operable bandwidth is within the range 0–2.5 Hz with minimal deviation upon the force estimation magnitudes and phase shifts. It is thus clear that the force sensor would be able to operate adequately within the frequency range found within a beating heart.

The force sensor’s repeatability was investigated using the setup for the dynamic loading. Here, an external force with known magnitude was cyclically applied upon the force sensor (at a rate of 0.25 Hz) for a given force range. The resulting linearity of the response, given as the *R* ^2^ value between the ground truth and estimated forces, was found to be *R* ^2^ = 0.953. The sensor’s repeatability, given as the standard deviation of the difference between the ground truth and estimated forces, was found to be 6.24 mN. These results clearly indicate the sensor’s high linearity and repeatable behaviour.

## 6 Conclusion and future work

This paper presented the design, development, and complete performance validation of a new type of catheter tip force sensor. The sensor’s working principle is based on a helical spring flexure. There are several benefits associated with the use of a helical spring flexural design as compared with other flexural designs, e.g., parallel hinged flexures combined with leaf springs (Gao et al., 2018) or multi-hinged and multi-component flexures (Tang et al., 2022). These include: 1) reduced number of flexural components, 2) simplicity of manufacturing and assembly, 3) simplicity of dimensioning, i.e., scalability, 4) larger flexural axi-symmetry, and 5) design based on known and well-established spring theory. The multi-component flexural design, as proposed in the designs of Gao et al. (2018); Tang et al. (2022), however, does provide the benefit of independent dimensioning and customization of the lateral and longitudinal flexures. In our proposed design, the helical spring flexure is combined with a FBG-inscribed MCF. The MCF is able to measure longitudinal strains which are proportional to the magnitudes of external longitudinal forces. Furthermore, the MCF is also able to measure curvature and it direction which is proportional to the magnitude and direction of external lateral forces. The advantage of using a MCF in the force sensor design not only allows for tip force sensing, but also shape sensing, body force estimation, and temperature monitoring. All of these capabilities can be integrated using one MCF within a single catheter embodiment. This paper further presents an elaborated approach to allow for the easy design and dimensioning of sensor parameters based on predetermined requirements. The approach provides a way to decouple between measurements related to combined longitudinal and lateral forces. Moreover, by using information from two extra unstrained FBG sets, the approach also provides a robust method to compensate for environmental temperature changes, nullifying their effect upon force estimations. An experimental force sensor prototype was constructed, calibrated, and its performance was validated with regards to several key aspects such as: *1)* force estimation accuracy, *2)* decoupling of longitudinal and lateral forces, *3)* input measurement *versus* output force linearity, *4)* temperature compensation performance, and *5)* dynamic performance. Targeted future work aspects include: *a)* design for medical sterilizability, *b)* inclusion of ablation electrodes and testing during ablation, and *c)* performing *in-vivo* animal experiments to study the feasibility of using the force sensor within a realistic environment.

## Data Availability

The original contributions presented in this work are included in the article/supplementary material. Further inquiries can be directed to the corresponding author.
